# Clinical practice guidelines for high-resolution breast PET, 2019 edition

**DOI:** 10.1007/s12149-021-01582-y

**Published:** 2021-01-25

**Authors:** Yoko Satoh, Masami Kawamoto, Kazunori Kubota, Koji Murakami, Makoto Hosono, Michio Senda, Masayuki Sasaki, Toshimitsu Momose, Kengo Ito, Terue Okamura, Keiichi Oda, Yuji Kuge, Minoru Sakurai, Ukihide Tateishi, Yasuhisa Fujibayashi, Yasuhiro Magata, Takeshi Yoshida, Atsuo Waki, Katsuhiko Kato, Teisuke Hashimoto, Mayuki Uchiyama, Seigo Kinuya, Tatsuya Higashi, Yasuhiro Magata, Akihiro Machitori, Hirotaka Maruno, Ryogo Minamimoto, Keiichiro Yoshinaga

**Affiliations:** 1 PET Nuclear Medicine Committee, Japanese Society of Nuclear Medicine, Tokyo, Japan; 2 Japanese Society of Nuclear Medicine, Tokyo, Japan; 3 Health Insurance Committee, Japanese Society of Nuclear Medicine, Tokyo, Japan; 4 Yamanashi PET Imaging Clinic, Shimokato 3046-2, Chuo City, Yamanashi Prefecture 409-3821 Japan

**Keywords:** Guidelines, Breast PET, FDG, Revised edition, Japanese society of nuclear medicine

## Abstract

Breast positron emission tomography (PET) has had insurance coverage when performed with conventional whole-body PET in Japan since 2013. Together with whole-body PET, accurate examination of breast cancer and diagnosis of metastatic disease are possible, and are expected to contribute significantly to its treatment planning. To facilitate a safer, smoother, and more appropriate examination, the Japanese Society of Nuclear Medicine published the first edition of practice guidelines for high-resolution breast PET in 2013. Subsequently, new types of breast PET have been developed and their clinical usefulness clarified. Therefore, the guidelines for breast PET were revised in 2019. This article updates readers as to what is new in the second edition. This edition supports two different types of breast PET depending on the placement of the detector: the opposite-type (positron emission mammography; PEM) and the ring-shaped type (dedicated breast PET; dbPET), providing an overview of these scanners and appropriate imaging methods, their clinical applications, and future prospects. The name “dedicated breast PET” from the first edition is widely used to refer to ring-shaped type breast PET. In this edition, “breast PET” has been defined as a term that refers to both opposite- and ring-shaped devices. Up-to-date breast PET practice guidelines would help provide useful information for evidence-based breast imaging.

## Introduction

High-resolution breast positron emission tomography (breast PET) imaging is a diagnostic imaging method with a PET scanner that has been specially designed for breast imaging. PET using ^18^F-2-deoxy-2-fluoro-D-glucose (FDG) is an extremely useful testing method for diagnosis and treatment. The FDG-PET examination has been covered by public health insurance since 2002 as a diagnostic imaging modality for 12 types of cancer, including breast cancer. Since then, PET has played an important role in the staging and diagnosis of breast cancer, including its recurrence [1].

Breast cancer has been the most common cancer in Japanese women since 1996, and both the number of cases and deaths have continued to increase [2]. Mammography is the only examination modality that has shown a mortality reduction effect, and has been introduced as a means of countermeasure-type screening for patients 40 years of age and older. However, mammography has a limited ability to detect breast cancer in younger and high-density breasts. In these cases, other examination modalities should be considered in addition to mammography.

In the treatment of breast cancer, breast-conserving surgery is a standard operative procedure. To select the correct surgical procedure, it is necessary to diagnose the extent of the pathological changes within the breast to achieve a negative surgical margin. It is also critical to detect any cancer that is present in the contralateral breast [3]. The application of breast reconstruction has increased in recent years, and determining whether the nipple and areola can be preserved is very important for the prevention of subsequent recurrence. Moreover, accelerated partial breast irradiation has been introduced, where a selected area of the breast, with accommodations, is irradiated after breast-conserving surgery, thus increasing the necessity of an accurate preoperative diagnosis of the extent of the cancer to determine adaptations and prevent recurrence [4]. Magnetic resonance imaging (MRI) has been useful for detecting the presence and extent of intra-mammary lesions with excellent sensitivity and specificity, but concerns have been raised that MRIs are affected by the menstrual cycle, have several side effects associated with the use of contrast media, and cannot be performed in patients with internal metals or claustrophobia.

Conventional whole-body PET demonstrates low sensitivity for small breast cancers of diameters of up to 1 cm. This may be due to the limited spatial resolution of whole-body PET devices, the decreased detectability of supine imaging due to respiratory movement, and the physiological FDG uptake in mammary glands. Even with scans in the prone position and delayed imaging, sufficient detectability cannot be currently obtained with a whole-body PET. Proximity-imaging breast PET scanners have been developed to overcome these challenges. As the breasts are located on the body surface, the detector can be brought as close as possible; thus, proximity imaging allows breast PET to obtain higher spatial resolutions than whole-body PET.

The detectors immobilize the breast to reduce respiratory movement, which has been successful in increasing detection sensitivity [5]. A detailed image of the distribution of FDG in the mammary gland can be obtained by scanning the breast for longer than the duration of a whole-body PET.

Between August 2012 and July 2018, three models of breast PET devices were approved in Japan, and the breast PET exam was included in the list of procedures covered by public health insurance in July 2013. Breast PET demonstrates approximately the same diagnostic performance as mammography and MRI, and is considered useful in the diagnosis and treatment of breast cancer. To be covered by public health insurance, it is required to be used in combination with conventional whole-body PET. This will enable more accurate staging and diagnosis of recurrence for breast cancer with a series examination of two PETs and it can greatly contribute to decisions regarding a patient’s treatment plan.

The first edition of these guidelines was published in July 2013 by the Japanese Society of Nuclear Medicine (JSNM) to provide a new compass for breast PET and facilitate its safe, smooth, and appropriate advancement. Since then, new types of breast PET scanners have also been used, and as these benefits became apparent, the need for new guidelines for breast PET has increased. For this reason, the guidelines have been revised. Additional revisions will be made as appropriate upon additional revisions of the devices, and as evidence warrants. Please note that the original Japanese document is the only valid version of this document, and this English translation is for reference only.

## Guidelines for personnel, equipment, and safety management

### Institutional standards

Breast PET is recommended in combination with whole-body PET or PET/CT. In addition, if the breast PET scan is covered by public insurance as a diagnostic imaging modality, then, given the whole-body PET scan requirement, the following facility standards regarding the calculation of medical payments must be fulfilled, as required for whole-body PET scans:

The presence of at least one full-time physician who has at least three years of experience in nuclear medicine diagnosis and has completed the specified training.

The presence of at least one full-time radiological technologist with experience and specialized knowledge in handling PET preparations for each diagnostic imaging device.

As with whole-body PET imaging, the presence of one breast PET device is presumed, and the presence of at least one full-time medical radiological technologist for each device is required. The rotation of multiple medical radiological technologists is also recommended from the perspective of occupational exposure involved in breast PET devices.

### Others

Regarding common points with whole-body PET examinations, such as imaging diagnosis and interpretation, institution-wide standards, imaging equipment maintenance and management, scanning methods, and safety management in PET scans, see the previous guidelines [1, 6, 7]

## Guidelines for breast PET scanners

### Scanner type

For breast PET, devices approved by the Pharmaceutical Affairs Law should be used. As of July 2018, there are two types of breast PET devices approved in Japan. Focusing on the arrangement of the detectors, these types will be referred to as the “opposite-type” and the “ring-shaped type.”

#### Opposite-type breast PET scanner

This scanner uses two flat plate-like detectors to image the breast, as in mammography. Direct immobilization of the breast can reduce the proximity of the detector and respiratory movement. Parallel tomographic images can be obtained by placing the breast between two detectors [8–10]. A series of four acquisitions in total, mediolateral oblique and cephalocaudal for the left and right breasts, is the standard.

#### Ring-shaped type breast PET scanner

This scanner features a ring-shaped (cylindrical) arrangement of detectors; the subject lies in the prone position, with the breast hanging in the center of the detector for imaging [11]. Two images are acquired, one for each breast.

### Inspection of scanner performance

#### When the equipment is installed and after large-scale repairs and adjustments

The spatial resolution and uniformity of the image density should be measured in accordance with the methods defined in the guidelines for prevention of adverse events in nuclear medicine from the JSNM. Comparisons are made with data on the same models published by the manufacturer and other institutions.

#### At the start of daily work

- Checking the general and operational status of the device.
- Checking the status of the detector.

#### Regular inspection and calibration

- General operation status of the device.
- Data acquisition for detector sensitivity correction.
- Correction of detector uniformity.

## Guidelines for breast PET imaging

These guidelines detail the applications of breast PET scans using FDG, the exam methods involved, and important points to consider in the diagnosis and image interpretation, from the perspective of nuclear medicine and breast imaging specialists.

### Breast PET examination covered by public medical insurance

#### Application of public health insurance

Insurance presumes that breast PET will be conducted in combination with whole-body PET. Insurance coverage is comparable to that for breast cancer in PET and PET/CT.

- Insurance coverage requirements

Breast PET is used in patients for whom staging and metastasis/recurrence diagnosis cannot be confirmed through other examinations or diagnostic imaging.

- b. Selection criteria for insurance coverage
  i) The patient must have a histopathologically confirmed malignancy.
  ii) If a definitive diagnosis cannot be made pathologically, the patient should be diagnosed with a high clinical probability of malignancy based on the clinical history, physical findings, diagnosis through imaging other than PET or PET/CT, tumor markers, or clinical observation.

*See “Uninsured use” for the use of breast PET in cases not covered by insurance.

#### Precautions regarding the subjects

- Since FDG uptake in the lactating mammary gland is higher than that in the non-lactating gland, lactating women should not be subjected to breast PET examination. In these cases, injection of FDG itself is not advisable and should be examined carefully only if the clinical benefit is determined to outweigh the adverse effects of exposure.
- If a PET scan has been used to diagnose malignancies other than breast cancer, the use of a breast PET scan for screening purposes should be avoided even in patients with a history of breast cancer. This guideline is designed to prevent the misuse of breast PET scans in insured treatment and does not apply to cases where breast cancer metastasis or recurrence is suspected. These guidelines are intended to prevent abuse of insured breast PET scans, and women suspected of having breast cancer metastasis or recurrence are not excluded.

### Examination protocol

#### FDG injection dose

FDG should be injected when the diagnostic benefit is deemed to outweigh the adverse effects of exposure. The FDG dose should be the minimum required.

Exposure doses are the same as those in whole-body PET scans, as breast PET scans generally do not require additional FDG injection. The doses presented in these guidelines are based on those reported in International Commission on Radiological.

Protection Publication 80 [12]. The effective dose when 185 MBq is applied to adults is 3.5 mSv, and the exposure by standard transmission scanning using a ^68^Ge-^68^ Ga source is approximately 0.25 mSv. On the other hand, the exposure from CT in PET/CT for attenuation correction is 1.4 to 3.5 mSv as low-dose CT for PET and CT fusion images. PET/CT should be carefully considered when CT imaging is at the same high dose as traditional diagnostic CT, as the exposure can exceed 10 mSv.

#### Scanning time

Whole-body PET imaging follows conventional guidelines. It is recommended that breast PET imaging be performed after whole-body PET imaging. If whole-body PET imaging is scanned 60–90 min after FDG injection, then breast PET should preferably be conducted at least 90 min after FDG injection. This is to reduce the exposure of the medical radiological technologist during breast imaging positioning, establish an appropriate imaging range with reference to whole-body PET images, and further increase the tumor-to-background ratio over time. The usefulness of delayed images of breast PET has not been established. An acquisition time of approximately 8 min per direction for opposite-type scanners and of approximately 5–10 min per breast for ring-shaped type scanners is recommended (with the optimal imaging time selected based on phantom experiments for each individual model).

#### Imaging position

In opposite-type scanners, the mediolateral oblique direction, in which almost the entire breast structure is imaged and has few blind areas, and the craniocaudal direction is scanned to complement the mediolateral oblique.

In the ring-shaped type scanners, each breast is scanned once in the prone/natural hanging position. The A/C (upper half region) of the breast is more likely to be out of the field of view than the B/D (lower half region); therefore, positioning should be performed such that the head is lowered and the chest wall is as close to the scanner as possible.

In both types of scanner, positioning should be performed with special attention in the following cases:

- Examination of pacemaker wearers

Care should be taken not to sever the pacemaker leads because of the body surface pressure exerted by the opposite- and ring-shaped type scanners.

b. Patients whose breasts have been surgically augmented with foreign bodies

As the breast is placed between the plate-like detectors in the opposite-type scanner, care must be taken to avoid damage to breast implants by detector compression.

### Image reconstruction and display

Each model has a default system for image reconstruction and display; the approach used is decided based on information from the manufacturer and other facilities using the same device models.

### Image storage and scan reports

The image data are generated as volume data with values. These data are transferred to and displayed on a computer attached to the imaging devices or to another computer. It is displayed as tomographic images in the appropriate directions, or as “maximum intensity projection images”, through display analysis software, as required by the diagnostic imaging physician. Image findings should always be recorded and returned to the requesting clinician in the form of a report. Preparation and handling of the report shall be performed in accordance with the methods specified in the Guidelines for the Prevention of Adverse Events in Nuclear Medicine, given by the JSNM.

The original image data must be stored in a digital format that can be retransmitted to the display analysis software. It should be noted that PET images may have incomplete DICOM conversion.

In addition, for ring-shaped type breast PET, mediolateral maximum intensity projection images, which are similar to mammographs, and three cross-sectional images, such as transverse, sagittal, and coronal, are recommended. It is also recommended to fix the monitor’s standardized uptake value (SUV) display range, as with whole-body PET, to reduce variability in diagnosis between readers.

### Determination and diagnosis

#### Visual assessment

In breast PET imaging, regions with higher FDG uptake than the background mammary glands are picked up by visual evaluation. It is recommended to compare these images with those obtained from mammography and breast ultrasonography as much as possible.

Even with high-resolution breast PETs, the FDG uptake of small lesions is affected by partial volume effects; therefore, not only the degree of accumulation (including the quantitative evaluation described below), but also the visual assessment is important. In particular, since the contrast effect of breast MRI images and the FDG uptake of breast PET images show similar findings, it is useful to evaluate the pattern and distribution of FDG uptake based on the interpretation method of contrast-enhanced breast MRI to distinguish between benign and malignant lesions [13].

#### Quantitative evaluation

The use of SUVs, which are also used in whole-body PET, is recommended for quantitative evaluation. SUV measurements are possible with ring-shaped type scanners, and they are known to correlate with whole-body PET SUVs [14].

In opposite-type scanners, unique quantitative parameters, such as the FDG accumulation ratio versus the dose per unit body weight (positron emission mammography standardized uptake value) and the lesion-to-background FDG uptake ratio, are used. Since the methods of attenuation correction differ between breast PETs and whole-body PET, the characteristics of the scanners must be considered in the quantitative assessment.

#### False negatives and false positives

Some malignancies with low glucose metabolism and low cellularity, such as non-invasive carcinomas and invasive lobular carcinomas, are difficult to detect with FDG-PET, which may result in false negatives. Attention must be similarly paid to this issue in breast PET.

In addition, FDG often accumulates in benign conditions, such as mastitis, local inflammation that occurs immediately after biopsy, so-called mastopathy, and intraductal papilloma, resulting in false positives. We must be aware that these accumulations can yield false-positive results that interfere with an accurate diagnosis.

Breast PET can detect smaller lesions due to its higher sensitivity and resolution compared to that of conventional whole-body PET. However, owing to the characteristic of FDG-PET as a glucose metabolic imaging modality, its ability to distinguish between benign and malignant tumors is limited.

### Clinical significance

#### In insurance coverage use

- Comparison with contrast-enhanced MRI

Currently, contrast-enhanced MRI is often used to diagnose the intra-mammary spread of primary breast cancer lesions. When the detection capabilities of breast PET and contrast-enhanced MRI are compared, the sensitivity is comparable, but the specificity is better for breast PET [15]. This is because contrast-enhanced MRIs yield more non-specific contrast enhancement (background parenchymal enhancement) in the background mammary glands of young women, whereas physiological FDG uptake in normal mammary glands in breast PET has less of an effect on lesion-to-background contrast [16]. In particular, breast PET is a good choice for patients who are not candidates for contrast MRI due to internal metal retention, claustrophobia, renal dysfunction, contrast media allergies, or other considerations.

b. Comparison of conventional modalities with PET for the staging and diagnosis of recurrence

The 2018 Breast Cancer Practice Guidelines [17] recommend PET/CT for advanced stage III and IV breast cancers regardless of age, as PET/CT is likely to detect unexpected metastatic disease. On the other hand, PET/CT is not recommended as a preoperative diagnostic exam for stage I and II breast cancers. In these cases, since the possibility of metastasis is low, the disadvantages of PET/CT (exposure, cost) may outweigh the benefits (detection of metastasis). However, patients aged < 40 years have a higher mortality rate [18], even for cancers diagnosed via conventional imaging at stages I and II, and a higher detection rate of distant metastasis with PET/CT [19]. This may correlate with the higher incidence rate of HER2-positive and triple-negative cancers in patients under 40 years [20].

#### FDG-PET as a predictor of prognosis

Correlations have been identified among the degree of FDG uptake in the primary breast cancer lesion prior to treatment and histopathological subtypes, prognosis (recurrence and mortality), and the St. Gallen risk categories [21, 22]. Pre-treatment PET is also useful for prognosis prediction.

#### MRI-guided biopsy and breast PET

In April 2018, MRI-guided breast biopsies were classified as an insurance-covered procedure based on the evidence that some breast cancers cannot be detected with conventional mammography or breast ultrasonography and can be visualized only with contrast-enhanced MRI. As described in “Visual assessment” positive images on contrast-enhanced MRI and breast PET often present similar findings; therefore, if legal issues, such as “Act on Prevention of Radiation Hazards due to Radioisotopes, etc.” are resolved in the future, it may be possible to perform breast PET-guided biopsy for breast PET-only positive lesions that cannot be visualized by conventional imaging.

#### Overdiagnosis

In recent years, along with advances in imaging, it has become possible to detect small breast cancers that had previously been difficult to identify. Breast PET will allow smaller breast cancers to be diagnosed. However, with breast cancer, it is also known that some lesions, such as certain non-invasive cancers, are not life-threatening, making the overdiagnosis of breast cancer an issue [23]. In the future, breast PET findings at the time of initial detection may be useful in determining the need for treatment; further case accumulation and consideration are expected.

### Uninsured use

#### Evaluation of post-chemotherapy effects in advanced breast cancer

The reduction rate in FDG uptake after the completion of two courses of chemotherapy in advanced breast cancer is considered to correlate with the histological evaluation of the treatment efficacy. If reproducibility is confirmed, an FDG-PET scan after two courses of chemotherapy can be used to determine whether chemotherapy with the same regimen should be continued [24–26]. The assessment of therapeutic efficacy of preoperative chemotherapy with breast PET has a higher accuracy rate than that with whole-body PET [27]; therefore, clinical benefits leading to improved prognoses are expected.

#### Voluntary breast cancer screening

The FDG-PET Cancer Examination Guidelines were prepared by the PET Detection Subcommittee of the Clinical PET Promotion Council in 2004. These guidelines aim to maintain standards for PET cancer screening, promote its sound development, and demonstrate its effectiveness. The guidelines were revised in 2007 based on the national survey data from Japan in 2005, with slight modifications added in 2011 due to the further integration of the JSNM's PET Nuclear Medicine Subcommittee, affiliated with the Clinical PET Promotion Council, and changes to PET cancer screening methods. During this period, a nationwide survey on PET cancer screening based on the guidelines was conducted in Japan from 2005 to 2009. The findings from the analysis of the survey were added, and the guidelines were revised for the 2019 edition.

The 2019 FDG-PET Guidelines for Cancer Screening [28] reported that FDG-PET had a sensitivity of 83.9% and a positive predictive value of 41.7% for detecting breast cancer, showing no significant differences between PET and PET/CT. Breast cancer foci are usually recognized as obvious FDG uptakes, but as with other cancers, small-sized lesions often have a low FDG uptake and may not be detected with PET; therefore, supplemental ultrasonography is required. Breast cancers with low cellularity (many scirrhous cancers) and well-differentiated cancers are cited as examples of sources of frequent false negatives.

Breast PET has permitted the detection of small breast cancers that are difficult to detect using conventional PET or PET/CT. Adding breast PET to health check-up examinations is expected to improve the breast cancer detection rate. At the same time, attention must be paid to the increase in the number of overdiagnoses.

#### Screening for high-risk groups

As morbidity increases, it has been recognized that genetic factors are associated with early-onset breast cancers, such as hereditary breast cancer and ovarian cancers, in which breast cancer susceptibility gene I (BRCA1) and breast cancer susceptibility gene II (BRCA2) play a role. These patients are characterized by a younger age of onset; hence, in them, breast cancer screening is recommended at an earlier age than usual. The American Cancer Society guidelines recommend MRI-based screening not only for BRCA1/2-positive women but also for those with a lifetime risk of greater than 20% for developing breast cancer [29–33]. Recent advances have shown that the rate of positive BRCA mutations in Japanese breast and ovarian cancer families is the same as that in Europeans and Americans. This has led to “Guidelines on breast MRI screenings for high-risk groups of breast cancer” [34]. Breast PET has the same level of diagnostic ability as that of contrast-enhanced MRI, and may be a useful systemic screening method when combined with whole-body PET. However, the basis for this assertion is not well understood at present, and it is therefore necessary to conduct clinical research involving a large number of cases and to examine the usefulness of breast PET.

## Radiation Safety

1. Basic concerns ensuring radiation safety

Radiation protection from the viewpoint of physical characteristics and radiation management in accordance with the law both conform to the standards followed in whole-body PET scans. In medical facilities that perform FDG-PET scans, taking into account social and economic factors, it is important to keep the radiation risk low within realistically achievable limits [35, 36].

2. Radiation safety management system

Follow the standards of whole-body PET scans.

3. Education and training of radiation workers

Follow the standards of whole-body PET scans.

4. Protection of medical staff associated with PET scanning from radiation exposure

The main radiation sources are the FDG injectable solution and the patients who receive it; therefore, it is necessary to follow the three principles of reducing external exposure (time, distance, and shielding). In breast PET scans, unlike whole-body PET scans, as the radiological technologist must touch the body (mainly the breasts) directly for positioning, increased exposure is unavoidable. Therefore, training is required to minimize the time taken for positioning. For breast PET scanners, which are directly connected to the control panel, scanning adjustments are carried out in the positron medical examination room; therefore, it is necessary to leave the examination room promptly once the imaging has started as well as to establish a system by which the progress of the examination can be checked. It is also recommended that more than one clinical radiological technologist be responsible for breast PET examinations and that duties be rotated among personnel.

5. Instructions and precautions for patients and patient nurses

Follow the standards of whole-body PET scans.

6. Structural and installation criteria

Follow the standards of whole-body PET scans.

7. Radiation waste disposal

Follow the standards of whole-body PET examination.

## Members of the revised breast PET clinical practice guidelines 2019

### Professional members

Koji Murakami, Masami Kawamoto, Kazunori Kubota, and Yoko Satoh.

### PET nuclear medicine committee

Chairperson: Makoto Hosono.

Vice Chairpersons: Michio Senda, Masayuki Sasaki, Toshimitsu Momose.

Members: Kengo Ito*, Terue Okamura, Keiichi Oda, Masami Kawamoto, Yuji Kuge, Minoru Sakurai, Ukihide Tateishi, Yasuhisa Fujibayashi*, Yasuhiro Magata, Koji Murakami, Takeshi Yoshida, and Atsuo Waki.

### Health insurance committee

Chairperson: Katsuhiko Kato.

Vice Chairperson: Teisuke Hashimoto.

Members: Kengo Ito, Mayuki Uchiyama, Seigo Kinuya, Tatsuya Higashi, Yasuhiro Magata, Akihiro Machitori, Hirotaka Maruno, Ryogo Minamimoto, Koji Murakami, Keiichiro Yoshinaga.

*Participated in drafting but not in approving the work in accordance with conflict of interest management standards.

## Members of the breast PET clinical practice guidelines, first edition, 2013

### PET nuclear medicine committee

Chairperson: Makoto Hosono.

Vice-chairperson: Tsuneo Saga.

Administrators: Kengo Ito, Shinichiro Kumita, Masayuki Sasaki, Michio Senda, Jun Hatazawa, Hiroshi Watanabe.

Committee members: Hiroshi Ito, Shinichi Kanaya, Yuichi Kimura, Hideo Saji, Seishi Jinnouchi, Hiroyoshi Fukukita, and Koji Murakami.

### Health insurance committee

Chairperson: Kengo Ito.

Vice-chairperson: Shinichiro Kumita.

Administrators: Seigo Kinuya, Junichi Yamazaki.

Committee members: Mayuki Uchiyama, Koichi Uno, Katsuhiko Kato, Tsuyoshi Kawano, Kazuo Kubota, Takashi Togawa, Norinari Honda, Hirotaka Maruno, Mana Yoshimura.

Guideline assistants: Masami Kawamoto, Yukihiko Ozawa.
